# Detection of Nociceptive Stimuli Using the Newborn Infant Parasympathetic Evaluation Index in Children Aged From 3 to 18 Years

**DOI:** 10.1111/pan.15129

**Published:** 2025-06-09

**Authors:** Frantisek Kolek, Jakub Jonas, Tomas Vymazal

**Affiliations:** ^1^ Department of Anaesthesiology and Intensive Care Medicine, Second Faculty of Medicine Charles University and Motol University Hospital Prague Czech Republic; ^2^ Faculty of Medicine Masaryk University Brno Czech Republic; ^3^ Emergency Medical Service ASCR Prague Czech Republic

**Keywords:** analgesia, newborn infant parasympathetic evaluation, patient comfort monitoring, pediatric, sedation

## Abstract

**Background:**

One option to objectively monitor patient stress response is to measure parasympathetic nervous system tone using respiratory arrhythmia analysis. The Newborn Infant Parasympathetic Evaluation (NIPE) Index has been developed for children younger than 2 years of age, and reliability has been confirmed by several studies.

**Aims:**

The aim of this study is to determine whether this method is also applicable to older children.

**Methods:**

Patients aged 3–18 years, admitted to the Department of Anaesthesiology and Intensive Care Medicine, Second Faculty of Medicine, Charles University and Motol University Hospital, Prague, were included in this study. NIPE monitoring was provided on patients with airways secured by endotracheal intubation or tracheostomy. NIPE values were recorded before endotracheal suctioning, 1 min after the start, and 5 min after the end of suctioning. Subsequently, the averages of the values were analyzed using ANOVA and the Scheffé test. Along with the NIPE value, changes in hemodynamic parameters were monitored during the suction, and the results of both methods were compared.

**Results:**

The NIPE value during endotracheal suctioning was significantly lower, with an average reduction of 13.4 points on a 100‐point scale, and returned to baseline 5 min after suctioning ended. No significant changes in hemodynamic parameters (heart rate and blood pressure) were observed, either in the whole group of patients or in the group not receiving catecholamine support.

**Conclusions:**

The NIPE index detects the stress response to endotracheal suctioning in children older than 3 years and is more sensitive than hemodynamic parameters, regardless of catecholamine therapy.

## 1 Introduction

Intensive care has always included analgosedation to ensure comfort for patients [1]. However, establishing an effective medication regimen for patients who cannot communicate can be challenging. Although several scoring systems are available for patient comfort monitoring, they tend to be subjective and require experienced medical staff [2]. In patients under muscle relaxation, the only observable manifestations of discomfort are signs of body stress response, often limited to tachycardia and hypertension—symptoms that may arise from various other causes. Therefore, several techniques have been developed in recent years to objectively assess patient stress, with outputs displayed as continuously measured numerical parameters on a monitor [3].

In clinical practice, devices that monitor either parasympathetic or sympathetic tone are already being used. This study utilized the measurement of respiratory arrhythmia as an indicator of parasympathetic tone. Two types of devices, each using slightly different mathematical algorithms, have been developed for use in intensive care. The first device, the Analgesia Nociception Index (ANI)—renamed the High Frequency Variability Index (HFVI) in 2022—is designed for monitoring adult patients. The second, the Newborn Infant Parasympathetic Evaluation (NIPE), is intended for children under 2 years of age. Both methods are based on the variability in the distance between consecutive QRS complexes on an ECG. The measurement output is a dimensionless number, where a value of 80 indicates maximum comfort, while lower values indicate increasing stress.

NIPE provides two modes of measurement. The first, ‘Current NIPE’, reflects a real‐time calculation based on the most recent 20 s of ECG recording. The second, ‘NIPE’, averages data over a 20‐min period, allowing for trend analysis over time. A target value above 55 is generally recommended, with values exceeding 80 indicating oversedation. The choice of which device to use for children older than 2 years remains uncertain. Some studies have demonstrated the reliability of ANI for older children [4, 5], while others confirm the ability of NIPE to detect nociceptive stimuli in children younger than 2 years and its routine use in some neonatal units [6, 7, 8, 9, 10]. However, data on the use of NIPE for older patients have not been published yet. The aim of this study is to determine whether this method is applicable to these children.

## 2 Materials and Methods

This monocentric, prospective, observational study was conducted in the Department of Anaesthesiology, Resuscitation and Intensive Care Medicine, Second Faculty of Medicine, Charles University and Motol University Hospital, Prague, Czech Republic, from May 2020 to December 2023. The study included 33 patients aged 3–18 years who required mechanical ventilation support and were admitted to our department, resulting in a total of 62 measurements.

Inclusion criteria for the study were (1) airways secured by endotracheal intubation or tracheostomy, (2) indication for endotracheal suctioning strictly based on the patient's medical condition, (3) high‐quality ECG signal during the measurement, and (4) sinus rhythm on the ECG. Exclusion criteria were (1) persistent signs of discomfort requiring an increase in analgosedation within 5 min after the suctioning, (2) circulatory instability requiring adjustments to catecholamine doses during the measurement intervals, (3) administration of medications affecting the parasympathetic nervous system (especially atropine), and (4) presence of a large pericardial effusion causing ECG abnormalities.

As this was an observational study, no additional devices were applied to the patient's body, no tissue samples were collected, and the measurements had no effect on the patient's treatment. Therefore, informed consent was not required. The research protocol was approved by the Ethics Committee for Multi‐Centric Clinical Trials of the Motol University Hospital (Reference No. EK‐239/20).

### 2.1 Study Protocol

Once the inclusion criteria were met and the NIPE monitor was available, the patient was enrolled in this study. The overall suction time was between 30 s and 2 min, according to the patient's needs. The current NIPE, blood pressure and heart rate were recorded in three time periods: ‘before value’ for values recorded just before suctioning, ‘suction value’ for values recorded 30–60 s after suctioning when the most pronounced changes in the variables were observed, and ‘after value’ for values recorded 5 min after the end of the suctioning.

### 2.2 Statistical Analysis

For data analysis, the Statistica 14.0 (TIBCO Software Inc.) program was used. Descriptive statistics were used to determine the mean, minimum and maximum values of current NIPE, heart rate and mean arterial pressure. The associations between current NIPE values and endotracheal suctioning, as well as between heart rate and mean arterial pressure, were analyzed using ANOVA and the Scheffé test. Given the potential impact of catecholamines on hemodynamic parameters, a subgroup analysis was conducted to compare patients with and without circulatory support.

## 3 Results

**FIGURE 1:**
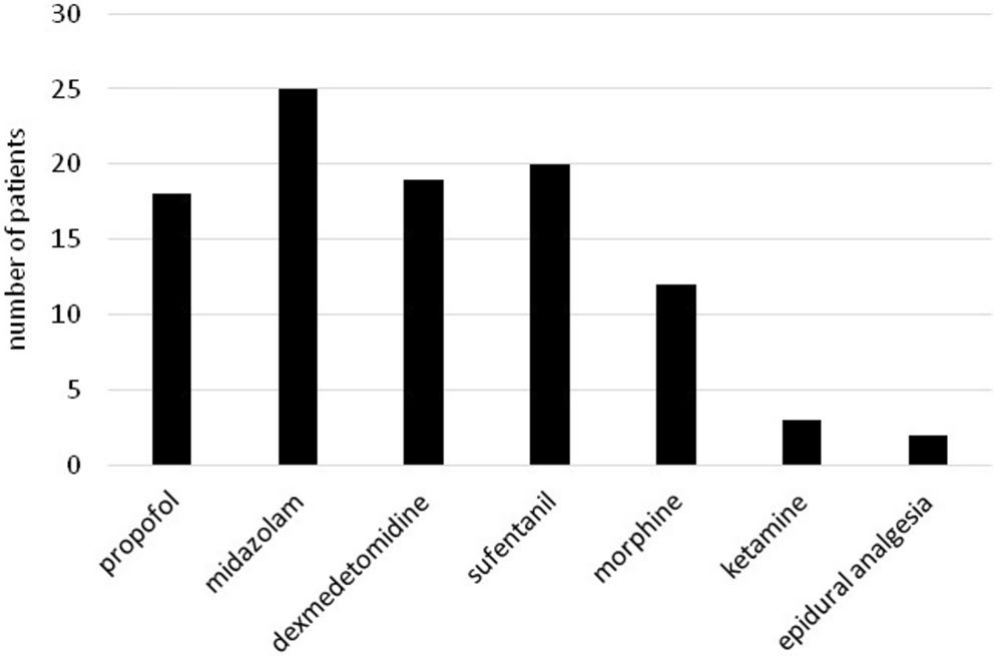
Use of analgosedation. Number of patients receiving different analgesic and sedative agents in the study population.

**FIGURE 2:**
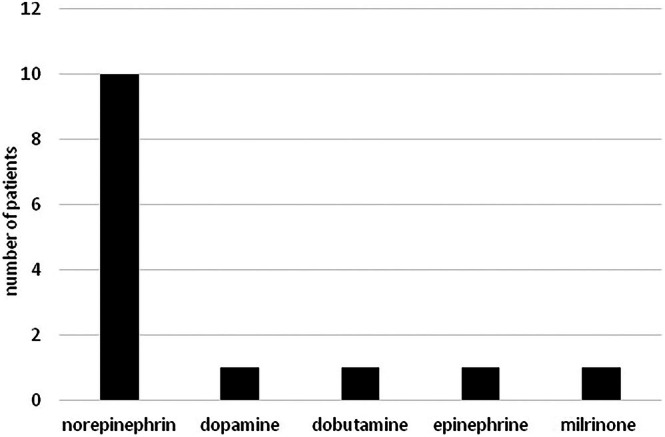
Use of circulatory support. Number of patients receiving different types of vasoactive and inotropic support in the study population.

**FIGURE 3:**
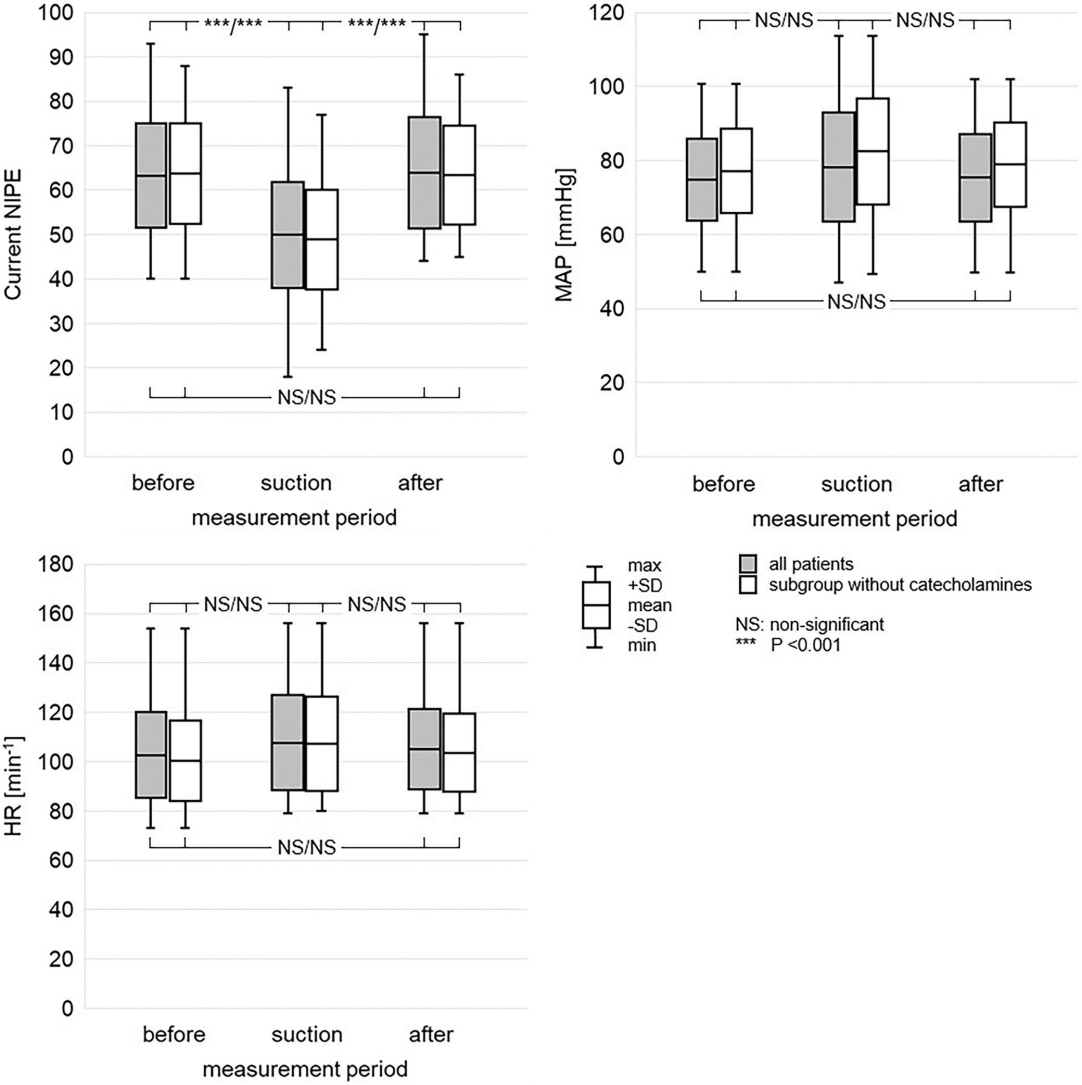
Current NIPE, mean arterial pressure and heart rate. Comparison of current NIPE, heart rate (HR) and mean arterial pressure (MAP) before, during, and after the endotracheal suctioning in all patients and in the subgroup of patients without any catecholamine support.

The study included 33 patients with a total of 62 measurements. The characteristics of the patients, including the etiology of the underlying disease, are shown in Table 1. The frequency of use of different types of sedatives and catecholamines is illustrated in Figures 1 and 2. The descriptive statistics of the measurements are shown in Table 2, and the graphical analysis of current NIPE, heart rate (HR), and mean arterial pressure (MAP) values is shown in Figure 3.

**TABLE 1:** Characteristics of patients.

	Mean (min; max) or *N* (%)
Age, years	9.4 (3; 18)
Catecholamine medication	
Yes	11 (33.3)
No	22 (66.6)
Reason for ICU admission	
Postoperative care	10 (30.3)
Sepsis	9 (27.3)
Unconsciousness	4 (12.1)
Trauma	3 (9.1)
Post‐resuscitation care	3 (9.1)
Respiratory failure	2 (6.1)
Liver failure	2 (6.1)

**TABLE 2:** Descriptive statistic data.

	Current NIPE	MAP, mmHg	HR, min^−1^
Before suction	63.3 ± 11.7 (40; 93)	74.8 ± 11.1 (50; 100.7)	102.6 ± 17.3 (73; 154)
During suction	49.9 ± 11.9 (18; 83)	78.2 ± 14.7 (47; 113.7)	107.7 ± 19.2 (79; 156)
After suction	63.9 ± 12.5 (44; 95)	75.4 ± 11.8 (49.7; 102)	105 ± 16.4 (79; 156)

*Note:* Data are shown as mean ± SD (min; max) of each parameter.

Abbreviations: HR, heart rate; MAP, mean arterial pressure; NIPE, Newborn Infant Parasympathetic Evaluation.

The mean current NIPE values were 63.3 ± 11.7, 49.9 ± 11.9, and 63.9 ± 12.5 before, during, and after suctioning, respectively. Statistically significant differences were found between current NIPE values during suctioning and both pre‐ and post‐suctioning values (both *p* < 0.001). However, no statistically significant difference was observed between current NIPE values before and after suctioning (*p* = 0.967). In the subgroup of patients not receiving catecholamines, mean current NIPE values were 63.8 ± 11.3, 48.9 ± 11.2, and 63.4 ± 11.2 before, during, and after suctioning, respectively. Similarly, statistically significant differences were found between current NIPE values during suctioning and both pre‐ and post‐suctioning values (both *p* < 0.001). However, no statistically significant difference was observed between current NIPE values before and after suctioning (*p* = 0.990).

The mean heart rate was 102.6 ± 17.3, 107.7 ± 19.2, and 105.0 ± 16.4 min^−1^ before, during, and after suctioning, respectively. No statistically significant differences were observed between heart rate during suctioning and both pre‐ and post‐suctioning values (*p* = 0.274, *p* = 0.696, respectively). Also, there was no statistically significant difference between heart rate before and after suctioning (*p* = 0.749). In the subgroup of patients not receiving catecholamines, mean heart rates were 100.2 ± 16.3, 107.3 ± 19.1, and 103.5 ± 15.8 min^−1^ before, during, and after suctioning, respectively. Similarly, in this subgroup, no statistically significant differences in heart rate were observed (*p* = 0.180, *p* = 0.617, *p* = 0.680).

The mean values of mean arterial pressure (MAP) were 74.8 ± 11.1, 78.2 ± 14.7, and 75.4 ± 11.8 mmHg before, during, and after suctioning, respectively. No statistically significant differences were observed between MAP during suctioning and both pre‐and post‐suctioning values (*p* = 0.332, *p* = 0.475, respectively). Also, there was no statistically significant difference between MAP before and after suctioning (*p* = 0.965). In the subgroup of patients not receiving catecholamines, mean MAP was 77.2 ± 11.4, 82.5 ± 14.3, and 78.9 ± 11.3 mmHg before, during, and after suctioning, respectively. Similarly, in this subgroup, no statistically significant difference in MAP was observed (*p* = 0.169, *p* = 0.431, *p* = 0.836).

## 4 Discussion

Establishing appropriate analgosedation for patients with secured airways can be challenging, even for experienced medical professionals [9]. This task becomes even more complex when muscle relaxants are administered. Inadequate sedation may lead to patient discomfort, which the patient is unable to communicate. On the other hand, excessive use of analgosedation can lead to tolerance and addiction, increasing the risk of severe withdrawal symptoms during analgosedation discontinuation [11, 12].

Measurement of sympathetic and parasympathetic system activity can provide valuable insights into patient stress response due to a discomfort. An ideal device would utilize data from parameters already measured as part of standard monitoring in intensive care units, eliminating the need for additional equipment. Traditionally, changes in blood pressure and heart rate were the primary measurable parameters for assessing the stress of sedated patients. However, reliance on hemodynamic parameters is inherently non‐specific, as these changes can be influenced by various factors, such as body temperature or the administration of antihypertensive medication.

To address these limitations, several devices have been developed to continuously measure changes in sympathetic and parasympathetic tone. Methods assessing sympathetic tone include pulse oximeter waveform analysis, although reference limits for pediatric patients have not been established yet [13]. Additionally, techniques such as psychogalvanic reflex measurement and pupillometry can be used in children but require the application of supplementary measuring equipment.

For parasympathetic tone measurement, heart rate variability analysis is commonly used. This method involves measuring the intervals between two QRS complexes over a 20‐s period to assess the power spectrum of heart rate variability, which is influenced by parasympathetic tone. In young children, this part of the spectrum is typically smaller than that observed in adults. Consequently, two instruments were developed: the ANI/HFVI for adult patients and the NIPE for children under 2 years of age, which focus on measuring changes in a narrower segment of the spectrum.

Theoretically, NIPE should be applicable for older children, while ANI might not function effectively in young children. However, data supporting this hypothesis remain unpublished. In our study, we demonstrate that NIPE can be used for children in the age range of 3–18 years, showing enhanced sensitivity compared to traditional monitoring of blood pressure and heart rate as indicators of stress.

The NIPE method presents an ideal solution for continuous monitoring of patient stress and sedation levels. However, it is important to note that changes in parasympathetic system tone not only reflect patient comfort but are also related to other conditions that activate the stress response. A typical example is sepsis, which leads to hyperactivity of the sympathetic system and a decrease in parasympathetic activity. Thus, a decrease in NIPE over several hours, despite adequate analgosedation and the exclusion of other sources of the stress, should raise suspicion of sepsis. This way, NIPE could contribute to early diagnosis and timely initiation of treatment for a serious infection [14]. Other factors can also influence NIPE measurements, including hemorrhage, which triggers a compensatory sympathetic response, and alterations in catecholamine dosage [15].

The limitations of NIPE include factors that can compromise the measurement of parasympathetic tone, such as the absence of sinus rhythm or poor ECG signal quality within the 20‐s measurement window. Additionally, medications that affect the parasympathetic nervous system, particularly atropine, and the presence of pericardial effusion can hinder accurate readings. Furthermore, NIPE cannot differentiate the specific source of the stress, which may arise from pain, cold sensations, or fear. Further studies using different types of pain stimuli or conducted in conscious patients would be necessary to validate the claim that NIPE can reliably detect the discomfort.

A limitation of this study is a small sample size. However, data from multiple measurements revealed statistically significant changes in the NIPE index, whereas changes in blood pressure and heart rate were not statistically significant.

## 5 Conclusion

In this observational study, we demonstrate that the Newborn Infant Parasympathetic Evaluation (NIPE) is an effective tool for monitoring parasympathetic tone in children aged 3–18 years, extending its application beyond the previously established limit of 2 years. Our findings indicate that the NIPE index serves as a more sensitive indicator of patient reactions to airway suctioning compared to traditional measures such as changes in blood pressure and heart rate.

NIPE is a strictly non‐invasive and relatively cost‐effective method, as it does not require any additional single‐use materials. A significant advantage of NIPE is its applicability in patients requiring muscle relaxation and in scenarios where standard scoring systems may not be feasible. Furthermore, NIPE can be utilized during the administration of catecholamines, enhancing its utility in clinical practice.

## Author Contributions

Frantisek Kolek designed the study conception, collected the data, and performed the statistical analyses. Frantisek Kolek and Jakub Jonas wrote the manuscript. Jakub Jonas and Tomas Vymazal reviewed the study conception. Tomas Vymazal reviewed the manuscript and contributed edits.

## Ethics Statement

The study was approved by the Ethics Committee for Multi‐Centric Clinical Trials of the Motol University Hospital (Reference No. EK‐239/20). The entire research was performed in accordance with the Declaration of Helsinki. Written informed consent was not required as this was an observational study; no additional devices were applied to the patient's body, no tissue samples were taken, the measurements had no effect on the patient's treatment, and no personal data were collected.

## Conflicts of Interest

The authors declare no conflicts of interest.

## Data Availability

All data analyzed during this study are included in this article. Requests for the complete dataset or further inquiries can be directed to the corresponding author (J.J.).
